# Alcohol‐Associated Liver Disease: Epidemiology, Pathogenesis, and Emerging Therapeutic Targets

**DOI:** 10.1002/mco2.71120

**Published:** 2026-11-05

**Authors:** Shuyan Wang, Yirui Hu, Huikuan Chu

**Affiliations:** ^1^ Division of Gastroenterology, Union Hospital, Tongji Medical College Huazhong University of Science and Technology Wuhan Hubei China

**Keywords:** alcohol‐associated liver disease, cell death, epidemiology, gut–liver axis, inflammation, microbiota, therapeutic targets

## Abstract

Alcohol‐associated liver disease (ALD) represents a leading cause of preventable liver‐related morbidity and mortality worldwide. Despite its growing global burden, no pharmacological therapies targeting disease progression have achieved clinical approval, with management still centered on alcohol abstinence and supportive care. Classic pathogenic mechanisms, including ethanol metabolism, oxidative stress, innate immune activation, and regulated cell death, are well recognized, yet emerging evidence highlights the gut–liver axis and intestinal dysbiosis as critical, interactive drivers of disease pathophysiology. However, how gut microbial imbalance mechanistically intersects with these canonical pathways to accelerate ALD progression remains insufficiently integrated. This article summarizes the epidemiology, clinical manifestations, and pathogenesis of ALD. Importantly, we focus specifically on changes in the gut microbiota (including bacteria, fungi, and viruses) and microbial metabolites. We further discuss the interactive relationships between dysbiosis and these core mechanisms. Finally, we summarize progress in preclinical and clinical research on targeted strategies such as probiotics, fecal microbiota transplantation, and phage therapy. By synthesizing the research findings, we propose a stage‐based, mechanism‐oriented framework aimed at advancing the development of safer and more precise therapeutic interventions for ALD.

## Graphical abstract

ALD imposes a growing global burden influenced by alcohol consumption, genetic susceptibility, and obesity. Its pathogenesis involves interactions among ethanol metabolism, oxidative stress, innate immune activation, regulated cell death, and disruption of the gut–liver axis. Microbiota‐directed strategies are now progressing from preclinical investigation to clinical evaluation and may contribute to future precision treatment.

Abbreviation: ALD, alcohol‐associated liver disease.

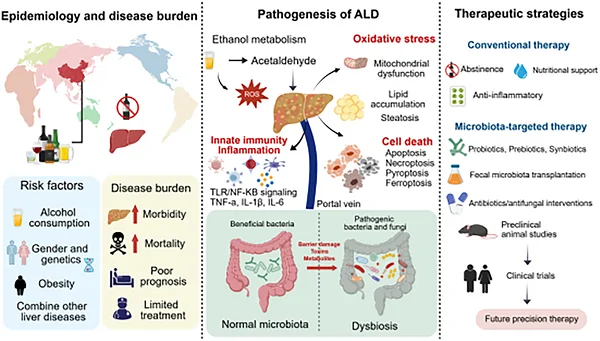

## 1 Introduction

Alcohol‐associated liver disease (ALD) is a major cause of chronic liver disease worldwide and has become a leading indication for liver transplantation [1, 2]. Harmful alcohol use caused approximately 2.6 million deaths in 2019—4.7% of all deaths globally—and alcohol accounts for 13% of all deaths among people aged 20–39 years [3]. Alcohol‐associated cirrhosis and hepatocellular carcinoma (HCC) together account for roughly 1% of all deaths worldwide, and their incidence continues to rise in most regions [4, 5]. This burden increased further during the coronavirus disease 2019 (COVID‐19) pandemic: between 2019 and 2020, hospitalizations for alcohol‐associated hepatitis (AH) in the USA rose by 15.6% despite an 8.7% decline in overall hospital admissions, with the sharpest increase among adults younger than 40 years [6].

ALD encompasses a well‐defined histological and clinical spectrum, although only a minority of heavy drinkers progress to advanced disease. Hepatic steatosis develops in more than 90% of individuals with sustained heavy consumption and is usually asymptomatic and reversible with abstinence [2, 7]. With continued drinking, 10–35% progress to alcohol‐associated steatohepatitis, characterized by hepatocyte ballooning, lobular neutrophilic infiltration, and pericellular fibrosis [2]. AH is the most severe clinical manifestation of this stage: it presents with acute jaundice and systemic inflammation and, in severe cases, carries a short‐term mortality of 30–50% [8, 9]. Persistent steatohepatitis drives progressive fibrosis; 8–20% of heavy drinkers ultimately develop cirrhosis, from which HCC arises at an annual incidence of approximately 1–2% [2]. Progression through this spectrum is neither uniform nor inevitable: the cumulative dose and pattern of drinking, sex, obesity and metabolic comorbidities, coexisting liver disease, and host genetic background all influence individual susceptibility, and every stage remains at least partially reversible upon sustained abstinence [3, 5]. Early recognition and intervention can therefore substantially alter the disease course [7, 9].

The pathogenesis of ALD results from interactions among direct hepatotoxicity, metabolic reprogramming, regulated cell death, and immune dysregulation [10]. Ethanol is oxidized by alcohol dehydrogenase and cytochrome P450 2E1 to acetaldehyde, generating reactive oxygen species (ROS) and toxic adducts, while suppression of peroxisome proliferator‐activated receptor‐α and activation of sterol regulatory element‐binding protein‐1c reprogram lipid metabolism toward steatosis [10]. Injured hepatocytes undergo multiple forms of regulated cell death—apoptosis, necroptosis, pyroptosis, ferroptosis, and the recently described cuproptosis—while mitochondrial dysfunction amplifies oxidative stress and impairs regeneration [11]. Alcohol‐induced gut dysbiosis and increased intestinal permeability expose the liver to pathogen‐associated molecular patterns, activating Toll‐like receptor 4 and the NLRP3 inflammasome in Kupffer cells and recruiting neutrophils and bone‐marrow‐derived monocytes. Epigenetic reprogramming, noncoding RNAs, and inherited susceptibility loci such as PNPLA3, TM6SF2, and MBOAT7 further influence the risk of progressive disease [10, 11, 12].

Therapeutic options remain limited. Abstinence is the cornerstone of management at every stage, yet pharmacotherapy for the underlying alcohol use disorder (AUD) is prescribed to fewer than 2% of patients with ALD, and relapse is common [1, 13]. For severe AH, corticosteroids are the only pharmacotherapy with proven short‐term benefit, but the landmark STOPAH trial showed that prednisolone improved 28‐day survival only modestly, without significant benefit at 90 days or 1 year, and increased serious infections (13 vs. 7%); approximately 40% of patients fail to respond, and nonresponders identified by a Lille score ≥0.45 fare particularly poorly [14, 15]. Pentoxifylline conferred no survival benefit. Early liver transplantation markedly improves survival in carefully selected steroid nonresponders but is constrained by organ scarcity, heterogeneous selection policies, and concerns about relapse [16]. Accordingly, recent clinical development has focused on several mechanism‐based approaches: agents targeting epigenetic regulation, liver regeneration, the gut–liver axis, and innate immunity have entered clinical testing, and several pivotal trials are underway [3, 16].

In this review, we provide an updated synthesis of the epidemiology of ALD; examine the cellular and molecular mechanisms that drive disease initiation and progression, from ethanol metabolism and lipid dysregulation to organelle dysfunction, regulated cell death, and gut–liver–immune crosstalk; and critically evaluate established and emerging therapeutic targets, with particular emphasis on agents now in clinical development.

## 2 Epidemiology

The epidemiology of ALD provides essential clinical context for mechanistic and therapeutic research because disease burden is shaped by population drinking patterns, socioeconomic factors, access to addiction care, comorbid obesity, viral hepatitis, and health‐system capacity [3, 17, 18]. Current epidemiological data show that ALD is both preventable and increasingly common, making early recognition and integrated intervention essential components of disease control [5, 19, 20].

### 2.1 Global and Regional Burden

ALD accounts for approximately 25–30% of cirrhosis‐related deaths and about 20% of liver‐cancer deaths worldwide, making alcohol a major cause of advanced liver disease [4, 5, 21]. The burden, however, is unevenly distributed. Europe historically reports the highest per‐capita alcohol consumption and the largest share of alcohol‐attributable liver mortality, with particularly high rates in Central and Eastern Europe, although age‐standardized death rates have begun to decline in several Western European countries following strengthened alcohol‐control policies [3, 5]. In the Americas, ALD is the leading cause of cirrhosis‐related mortality, and it has become the fastest‐growing indication for liver transplantation in the USA, surpassing chronic hepatitis C [5, 22]. The Asia‐Pacific region, historically considered a lower‐burden region, now shows the steepest growth: the Western Pacific subregion records the highest age‐standardized incidence rates, driven by rapid urbanization, rising incomes, and increasing obesity prevalence [23]. In China, a pooled analysis estimated the prevalence of ALD at 4.8%, and in a nationwide cohort of 136,571 patients with chronic liver disease, alcohol‐associated disease was among the strongest predictors of HCC development [24, 25].

Severe AH, although less prevalent than cirrhosis, contributes disproportionately to mortality and healthcare utilization. In the USA, AH hospitalizations rose steadily through the 2010s and accelerated during the COVID‐19 pandemic, reaching 157,885 admissions in 2020—a 15.6% increase over 2019—with a 23% increase among men younger than 40 years and markedly higher inpatient mortality when complicated by COVID‐19 infection (11.4 vs. 4.1%) [6]. These data indicate a demographic shift, with ALD increasingly affecting young adults [3].

### 2.2 Trends, Projections, and Risk Factors

Temporal analyses of Global Burden of Disease data reveal divergent trajectories: age‐standardized death rates of ALD are declining in much of Europe and Latin America, whereas age‐standardized incidence rates continue to climb across most of the Asia‐Pacific region—a pattern projected to persist through 2040 [23, 26]. Mortality is also shifting toward younger ages, and the gap between male and female alcohol consumption is narrowing [3, 26].

Modeling studies project a substantial increase in the ALD burden over the next two decades. In the USA, the prevalent ALD population is projected to grow from 20.3 million in 2019 to 29.1 million by 2040 (a 43% increase), with decompensated cirrhosis cases rising by 77% and annual deaths increasing by 79%, from 111,300 to 199,500; associated healthcare costs are expected to more than double [27, 28]. A parallel increase is projected among older adults globally, in whom ALD deaths are expected to rise from 16,187 in 2019 to 27,394 by 2040 (a 69% increase), with the 65–74‐year group growing fastest [29]. In the Asia‐Pacific region, incidence is projected to keep rising through 2040 even as age‐standardized mortality declines modestly, implying a growing prevalent population requiring long‐term care [23]. These projections strengthen the need for effective prevention and treatment.

Susceptibility to ALD is determined by interacting environmental and host factors. Risk rises steeply with the dose, duration, and pattern of drinking—particularly above approximately 20–30 g/day in women and 30–40 g/day in men—while binge drinking and drinking between meals confer additional risk independent of total intake [1, 4]. Women develop advanced ALD at lower cumulative doses and progress more rapidly than men, reflecting lower gastric alcohol dehydrogenase activity, differences in body composition, and hormonal factors, and alcohol consumption among women is rising in many countries [3, 9]. Obesity and metabolic dysfunction interact with alcohol—heavy drinkers with obesity carry a several‐fold higher risk of cirrhosis than those with either exposure alone—and the overlapping entity MetALD is increasingly recognized; the global rise in obesity is expected to amplify the future ALD burden [8, 10, 23]. Coexisting chronic liver disease, particularly chronic viral hepatitis (HBV or HCV), accelerates fibrosis and increases HCC risk in heavy drinkers [1, 5]. Genetic susceptibility further modulates risk: the PNPLA3 rs738409 (I148M) variant is the best‐established common risk allele, conferring as much as a threefold increase in cirrhosis risk in meta‐analyses, with additive effects in heavy drinkers, and genome‐wide studies have additionally implicated TM6SF2 and MBOAT7 as risk loci and HSD17B13 loss‐of‐function as protective, as discussed mechanistically in Section 3.3 [11, 12]. Tobacco use independently accelerates fibrosis progression and increases HCC risk in patients with ALD, and lower socioeconomic status is associated with disproportionately higher alcohol‐attributable harm for the same level of consumption—the “alcohol harm paradox”—reflecting clustered risk behaviors and reduced access to care [1, 3, 9]. Recognizing these factors has direct clinical value: it identifies high‐risk drinkers for targeted screening, informs stratification in clinical trials, and links ALD prevention to the broader metabolic‐health agenda.

## 3 Pathogenesis of ALD

ALD pathogenesis involves an interconnected network of cellular and interorgan processes. Recent studies have emphasized the importance of these interactions in disease progression [30]. In this network, ethanol metabolism initiates hepatocellular stress, gut barrier failure supplies microbial ligands, and innate immune responses convert injury into self‐amplifying inflammation [2, 31]. The following subsections emphasize mechanisms with strong translational relevance and distinguish established human evidence from hypotheses mainly supported by experimental models.

### 3.1 Ethanol Metabolism, Oxidative Stress, and Lipotoxicity

Ethanol is oxidized primarily to acetaldehyde by alcohol dehydrogenase and cytochrome P450 2E1 (CYP2E1), and acetaldehyde is subsequently converted to acetate by aldehyde dehydrogenase [4, 32, 33]. Excessive ethanol exposure increases the hepatic NADH/NAD+ ratio, suppresses fatty acid oxidation, promotes triglyceride accumulation, and alters mitochondrial respiration [33, 34]. CYP2E1 induction generates ROS and lipid peroxidation products that damage mitochondrial DNA, endoplasmic reticulum function, and cellular membranes [35]. Acetaldehyde forms protein and DNA adducts that impair protein trafficking, promote immune recognition, and contribute to fibrogenesis [32, 33].

Nonoxidative ethanol metabolism also generates bioactive lipids such as phosphatidylethanol and fatty acid ethyl esters, which can disrupt membrane structure and mitochondrial energy production [6, 36]. These products have diagnostic value as biomarkers of alcohol exposure and may also participate in organ injury beyond the liver [36]. Alcohol also modifies methionine metabolism, glutathione availability, lipid droplet turnover, and autophagy, creating a metabolic environment that sensitizes hepatocytes to inflammatory and microbial stimuli [37, 38]. Thus, ethanol metabolism is necessary but insufficient to explain severe ALD; its pathological impact depends on interaction with immune activation, gut‐derived signals, and impaired repair [31, 39].

### 3.2 Innate Immunity, Cytokines, and Inflammatory Signaling

Alcohol‐induced liver injury activates innate immunity through DAMPs from injured hepatocytes and PAMPs from the intestine [2, 31]. Kupffer cells respond to lipopolysaccharide (LPS), bacterial DNA, extracellular ATP, HMGB1, and other danger signals by activating NF‐κB, MAPK, interferon regulatory factors, and inflammasome pathways [40]. These pathways increase the production of tumor necrosis factor‐α, interleukin (IL)‐6, IL‐1β, IL‐18, chemokines, and ROS, which recruit neutrophils and monocytes and further injure hepatocytes [11, 41]. Neutrophils are a histological hallmark of AH, yet their function may be paradoxical because they contribute to tissue damage while patients remain susceptible to bacterial and fungal infections [42].

ALD is characterized by dysregulated rather than simply excessive immune activity. Patients with severe AH often display simultaneous systemic inflammation and immune paralysis, which helps explain why corticosteroids may reduce inflammatory mortality in selected patients while increasing infection risk in others [43]. Animal models can reproduce components of ethanol‐induced steatosis, gut leakiness, and inflammatory cytokine release, but they do not fully reproduce human AH with jaundice, severe fibrosis, infection susceptibility, and multiorgan failure [39]. For this reason, mechanistic conclusions based on mice should be interpreted alongside human transcriptomic, histological, and clinical data [44].

### 3.3 Genetic Susceptibility

Host genetic variation substantially influences susceptibility to ALD and helps explain why only a minority of heavy drinkers progress to advanced disease. Variants in alcohol‐metabolizing genes, particularly ADH1B and ALDH2, alter ethanol clearance and acetaldehyde exposure and thereby modify both drinking behavior and liver injury risk. Among lipid‐associated loci, the PNPLA3 rs738409 (I148M) variant is the best‐established common risk allele: meta‐analyses associate it with both predisposition to and severity of ALD, conferring as much as a threefold increase in cirrhosis risk with additive effects in heavy drinkers [11]. Genome‐wide association studies have confirmed PNPLA3 and additionally identified TM6SF2 (E167K) and MBOAT7 as risk loci for alcohol‐related cirrhosis, whereas HSD17B13 loss‐of‐function variants attenuate liver injury [12]. These loci converge on hepatocellular lipid handling—lipid droplet remodeling, very‐low‐density lipoprotein secretion, and retinol metabolism—linking genetic risk to the steatotic and lipotoxic phenotype described in Section 3.1. The marked interindividual variability in disease severity, histological progression, and clinical outcomes despite equivalent alcohol exposure therefore partly reflects genetic variation, and polygenic risk profiling may eventually refine risk stratification and trial enrollment [2, 12].

### 3.4 Epigenetic and Noncoding RNA Regulation

Epigenetic remodeling—encompassing DNA methylation, histone modifications, and noncoding RNA networks—regulates hepatic inflammation, innate immunity, oxidative stress, and fibrogenic signaling without altering the genomic sequence. Chronic alcohol exposure reshapes DNA methylation and histone acetylation patterns in hepatocytes and immune cells, altering the expression of genes involved in lipid metabolism and inflammatory responses [45]. Among noncoding RNAs, microRNAs are the best characterized in ALD: alcohol dysregulates hepatocyte miR‐148a through FoxO1, facilitating TXNIP overexpression and pyroptotic injury, and miR‐122 and miR‐155 link alcohol exposure to lipid dysregulation and innate immune activation [46]. Long noncoding RNAs and circular RNAs—more extensively studied in metabolic dysfunction‐associated steatotic liver disease (MASLD), where they modulate de novo lipogenesis, fatty acid β‐oxidation, lipotoxicity, oxidative stress, and fibrogenesis in hepatocytes, hepatic stellate cells, and Kupffer cells—are increasingly implicated in steatotic liver disease progression [47]. Because epigenetic marks are reversible and cell‐type specific, they represent plausible biomarkers and therapeutic targets, although most evidence in ALD remains associative or derived from experimental models.

### 3.5 Mitochondrial Dysfunction

Mitochondrial dysfunction amplifies ALD pathogenesis by disrupting cellular bioenergetics and redox homeostasis. Ethanol metabolism induces mitochondrial DNA damage, uncouples oxidative phosphorylation, and increases electron leak‐derived ROS, while impaired PINK1/Parkin‐mediated mitophagy permits the accumulation of dysfunctional mitochondria [33, 45]. Defective mitochondrial quality control extends beyond canonical mitophagy: mitochondria–lysosome‐related organelles and other lysosomal clearance routes participate in damaged‐mitochondria disposal, and their failure promotes hepatocyte dedifferentiation in chronic liver disease, including AH [33]. Consequently, impaired ATP synthesis, excessive ROS release, and lipid peroxidation lower the threshold for hepatocyte apoptosis and necroptosis, creating a self‐perpetuating cycle that drives disease progression from simple steatosis to advanced fibrosis, cirrhosis, and HCC [7].

### 3.6 Regulated Cell Death

Hepatocyte death is a central event in ALD because it converts metabolic stress into inflammation, fibrosis, and regenerative demand [45, 48, 49]. Unlike accidental necrosis, regulated cell death follows genetically encoded, pharmacologically tractable programs whose initiation and execution are shaped by ethanol metabolism, oxidative stress, and organelle damage. Apoptosis, necroptosis, ferroptosis, pyroptosis, and autophagy‐related processes can coexist in alcohol‐exposed livers, and their relative contribution likely changes with disease stage and injury context [50, 51]. Importantly, these modalities are not isolated: they share upstream stress signals, cross‐regulate one another, and can compensate for each other when a single route is blocked, which partly explains why single‐pathway interventions have yielded only partial protection in preclinical models [48, 52]. The following subsections summarize the defining features and ALD‐specific evidence for each major cell‐death pathway.

#### 3.6.1 Apoptosis

Apoptosis proceeds through intrinsic mitochondrial (Bax/Bak‐dependent cytochrome *c* release and Caspase‐9/‐3 activation) or extrinsic death‐receptor cascades, culminating in phosphatidylserine exposure and nuclear fragmentation [49, 50, 51]. In ALD, chronic ethanol exposure primes hepatocytes for apoptosis through CYP2E1‐derived oxidative stress, mitochondrial membrane depolarization, endoplasmic reticulum stress, and upregulation of death receptors and their ligands, and the extent of hepatocyte apoptosis correlates with disease severity in patients with AH [2, 50]. Although apoptosis is morphologically silent, apoptotic bodies that escape efferocytosis undergo secondary necrosis and release damage‐associated molecular patterns (DAMPs), thereby feeding hepatic inflammation [49]. Pharmacological caspase inhibition reduces hepatocyte apoptosis and liver injury in experimental ALD, but broad caspase blockade has shown limited benefit and safety concerns in clinical translation, suggesting that apoptosis is best targeted indirectly through its upstream stressors [48].

#### 3.6.2 Necroptosis

Necroptosis requires RIPK1/RIPK3 phosphorylation‐dependent MLKL oligomerization and plasma membrane translocation, resulting in lytic rupture and DAMP release [2]. Hepatic RIPK3 expression is markedly upregulated in patients with AH and in ethanol‐fed mice, and genetic RIPK3 deficiency or pharmacological RIPK1 inhibition attenuates ethanol‐induced steatosis, inflammation, and hepatocyte death in experimental models [52]. Unlike apoptosis, necroptosis is inherently proinflammatory: the ruptured plasma membrane releases HMGB1, ATP, and other DAMPs that activate Kupffer cells and recruit neutrophils, coupling cell death to the amplification of innate immune responses characteristic of severe AH [49, 50]. Because Caspase‐8 activity normally restrains necroptotic signaling, the balance between apoptotic and necroptotic pathways may determine whether hepatocyte loss remains relatively silent or drives inflammatory amplification [48, 52].

#### 3.6.3 Ferroptosis

Ferroptosis is executed by iron‐catalyzed lipid peroxidation upon GPX4 inactivation, a process amplified by alcohol‐driven oxidative stress and hepatic iron overload [44, 53]. The liver is particularly susceptible in ALD: chronic alcohol consumption promotes intestinal iron absorption and hepatic iron deposition, depletes glutathione, and suppresses the system Xc−–GPX4 axis, while CYP2E1‐derived ROS continuously peroxidize membrane polyunsaturated phospholipids [50, 53]. Ferroptotic hepatocytes release lipid peroxidation products and DAMPs that activate hepatic stellate cells and macrophages, thereby linking ferroptosis to fibrogenesis and sustained inflammation [44]. In experimental ALD, ferroptosis inhibitors such as Ferrostatin‐1 and iron chelators attenuate liver injury, and natural compounds including ursolic acid protect in part by restoring GPX4‐dependent antioxidant defenses, supporting ferroptosis as a tractable therapeutic target [54].

#### 3.6.4 Pyroptosis

Pyroptosis is a lytic and proinflammatory form of regulated cell death mediated by gasdermin pore formation, most classically downstream of inflammasome activation and Caspase‐1 cleavage of gasdermin D (GSDMD) [55, 56, 57]. Noncanonical pyroptosis is triggered when cytosolic LPS activates human Caspase‐4/5 or murine Caspase‐11, leading to GSDMD cleavage, potassium efflux, NLRP3 activation, and IL‐1β/IL‐18 release [58, 59]. In experimental ALD, Caspase‐11/4‐GSDMD signaling, NLRP3 activation, TXNIP upregulation, and hepatocyte mitochondrial dysfunction have all been implicated in pyroptotic injury [60, 61, 62]. Kupffer cell pyroptosis can amplify cytokine production, whereas hepatocyte pyroptosis directly releases DAMPs that activate nonparenchymal cells and recruit inflammatory leukocytes [63].

Pyroptosis is an attractive therapeutic target, although its clinical relevance remains uncertain. Genetic or pharmacological suppression of NLRP3, Caspase‐1/11, GSDMD, or upstream oxidative stress improves liver injury in several models, but complete inflammasome inhibition could impair antimicrobial defense and tissue repair [64, 65, 66]. Human evidence supports activation of pyroptosis‐related pathways in AH, yet causal proof and stage‐specific cell‐type resolution remain limited [57, 61, 62]. Current evidence therefore supports pyroptosis as an important amplifier of ALD inflammation rather than the sole disease mechanism [35, 67, 68].

#### 3.6.5 Autophagy

Autophagy maintains homeostasis via LC3‐II‐mediated autophagosome formation and lysosomal turnover of damaged mitochondria, whereas defective mitophagy or excessive autophagic flux can shift this response toward hepatocyte demise in ALD [50]. Recent hepatocyte‐specific knockout studies show that loss of autophagy‐related protein 14 simultaneously activates apoptosis, necroptosis, and NLRP3‐mediated pyroptosis, indicating that basal autophagy restrains multiple regulated cell death pathways in the liver [68]. The autophagy receptor SQSTM1/p62 is required for Mallory‐Denk body formation, which appears to act as an adaptive protective response to alcohol‐induced proteotoxic stress rather than a purely pathogenic one [69].

### 3.7 Gut–Liver Axis

#### 3.7.1 Alcohol‐Induced Bacterial Dysbiosis

Human and animal studies consistently show that alcohol exposure changes the composition and function of gut bacterial communities, although the direction of specific taxonomic shifts is not always identical across populations, sequencing platforms, disease stages, and medication exposures [29, 70, 71, 72]. Reported changes in bacterial communities in ALD are summarized in Table 1. In patients with AUD, AH, or cirrhosis, studies have reported enrichment of *Enterococcus*, *Streptococcus*, *Veillonella*, *Proteobacteria*, and oral‐derived taxa, with depletion of *Akkermansia*, *Faecalibacterium*, *Roseburia*, *Lachnospiraceae*, *Ruminococcaceae*, and other butyrate‐producing organisms [16, 73]. *Streptococcus* abundance has been associated with liver injury severity, while fecal microbial profiles and metabolic pathways have improved short‐term mortality prediction in AH beyond conventional clinical scores in some studies [44, 46, 74]. Cytolysin‐positive *Enterococcus faecalis* is of particular interest because it is associated with increased mortality and can exacerbate liver injury in humanized mouse models [24, 28, 75].

**TABLE 1:** Changes in bacterial communities in ALD.

References	Participants	Method	Genus	Species
Chen et al. [76]	HC (*n* = 24); patients with cirrhosis (*n* = 36)	454 pyrosequencing of the 16S rRNA V3 region and real‐time quantitative PCR	*Staphylococcus spp*.↑ *Streptococcus*↑ *Veillonella*↑ *Coprococcus*↓ *Roseburia*↓ *Pseudobutyrivibrio*↓	*Escherichia coli*↑
Dubinkina et al. [29]	Patients with alcohol dependence and cirrhosis (*n* = 27); patients with alcohol dependence without cirrhosis (*n* = 72)	Metagenomic analysis of fecal microbiota	*Lactobacillus spp*.↑ *Bifidobacterium*↑	
Llopis et al. [77]	Patients without AH (*n* = 16); patients with nonsevere AH (*n* = 12); patients with severe AH (*n* = 10)	Gut microbiome sequencing	*Streptococcus*↑ *Bifidobacteria*↑	
Grander et al. [78]	HC (*n* = 16); patients with nonsevere AH (*n* = 21); patients with severe AH (*n* = 15)	Fecal mucosal microbiota analysis	*A. muciniphila*↓	
Bajaj et al. [9]	HC (*n* = 43); patients with cirrhosis (*n* = 43)	Analysis of stool microbiome composition and function	*Bacteroides*↓	
Lang et al. [49]	AH with bilirubin ≤14.1 mg/dL (*n* = 37); AH with bilirubin >14.1 mg/dL (*n* = 36)	16S rRNA gene sequencing of fecal samples from patients with AH	*Veillonella*↑ *Enterococcus*↑ *Akkermansia*↓	
Tuomisto et al. [18]	HC (*n* = 7); patients with alcohol‐associated cirrhosis (*n* = 13); patients with alcohol dependence without cirrhosis (*n* = 15); nonalcoholic controls (*n* = 14)	Real‐time quantitative PCR (RT‐qPCR) of major commensal bacterial communities	*Gram‐negative Bacteroides spp*.↑ *Gram‐negative Enterobacter spp*.↑	
Duan et al. [28]	Participants without AUD (*n* = 26); patients with AUD (*n* = 44); patients with AH (*n* = 88)	Fecal 16S rRNA sequencing, Enterococcus culture, and qPCR		*Enterococcus faecalis*↑
Zhong et al. [74]	HC (*n* = 27); patients with alcohol‐associated fatty liver (*n* = 21); patients with alcohol‐associated cirrhosis (*n* = 17)	Fecal 16S rRNA sequencing	*Faecalibacterium*↓ *Lachnospira*↓ *Agathobacter*↓ *Ruminococcus_1*↓ *Fusobacterium*↑ *Escherichia‐Shigella*↑ *Streptococcus*↑ *Veillonella*↑	
Kim et al. [46]	HC (*n* = 24); patients with severe AH (*n* = 24)	16S rRNA amplicon sequencing and metagenomic analysis	*Veillonella*↑ *Oscillibacter*↓ *Eubacterium_g23*↓	
Addolorato et al. [1]	HC (*n* = 36); patients with AUD (*n* = 36)	Analysis of stool microbial composition and gut microbial function	*Sutterella*↑ *Haemophilus*↑ *Staphylococcus*↑ *Paraprevotella*↑ *Eubacterium*↑ *Streptococcus*↑ *Odoribacter*↑ *Veillonella*↑ *Enterococcus*↑ *Lactobacillus spp*.↑ *Akkermansia*↓ *Blautia*↓ *Bifidobacterium*↓ *Coprococcus*↓ *Dorea*↓ *Anaerostipes*↓ *Adlercreutzia*↓ *Ruminococcus*↓	
Shao et al. [73]	HC (*n* = 47); patients with compensated cirrhosis (*n* = 49); patients with decompensated cirrhosis (*n* = 46)	Ultra‐performance liquid chromatography–mass spectrometry	*Alistipes*↓ *Odoribacter*↓ *Eubacterium*↓ *Ruminococcus*↓ *Tannerella*↓ *Bilophila*↓ *Haemophilus*↑ *Megasphaera*↑ *Lactobacillus*↑ *Streptococcus*↑ *Veillonella*↑	

Several trends emerge from the bacterial literature. First, dysbiosis is not merely an epiphenomenon of cirrhosis because transfer of microbiota from susceptible patients can increase intestinal permeability and liver injury in recipient mice [75, 77]. Second, disease‐associated taxa may contribute through distinct mechanisms, including toxin production, endogenous ethanol generation, LPS enrichment, bile acid transformation, and loss of protective metabolites [79, 80, 81, 82]. Third, medication and host factors can confound microbial signatures; for example, proton pump inhibitor use promotes *Enterococcus* overgrowth and worsens alcohol‐induced liver injury in experimental models [83]. Therefore, future clinical microbiome studies should stratify by disease stage, medication exposure, nutrition, alcohol abstinence status, and coexisting metabolic disease [26, 46, 84].

#### 3.7.2 Alcohol‐Induced Fungal and Viral Dysbiosis

Nonbacterial components of the gut ecosystem have become increasingly prominent in ALD research. Reported changes in fungal communities in ALD are summarized in Table 2. Fungal dysbiosis in ALD is characterized by reduced fungal diversity and expansion of *Candida* species, and systemic antifungal immune responses correlate with clinical severity and mortality in AH [32, 50]. *Candida albicans* produces candidalysin, a peptide toxin that can directly damage hepatocytes and promote liver injury, providing a mechanistic link between the mycobiome and ALD and supporting a potential causal role [72, 85, 86]. *Candida*‐specific Th17 responses further suggest that adaptive immune responses to fungi participate in liver inflammation and may help stratify disease severity [87]. Fungal signatures may also help distinguish ALD from MASLD, supporting the diagnostic potential of multikingdom microbiome profiling [35, 88].

**TABLE 2:** Changes in fungal communities in ALD.

References	Participants	Method	Genus	Species
Chu et al. [22]	HC (*n* = 11); patients with AUD (*n* = 42); patients with AH (*n* = 91)	Analysis of C. albicans and ECE1 in fecal samples		*C. albicans↑*
Hartmann et al. [89]	HC (*n* = 18); patients with AUD (*n* = 66)	ITS2 amplicon sequencing of fecal samples	*Candida↑* *Debaryomyces↑* *Pichia↑* *Kluyveromyces↑; Issatchenkia↑* *Scopulariopsis↑* *Issatchenkia orientalis↑* *Scopulariopsis cordiae↑* *Aspergillus↑* *Kazachstania humilis↑*	*C. albicans↑* *C. zeylanoides↑*
Faria et al. [37]	Patients with severe AH receiving corticosteroid therapy (*n* = 6); patients with severe AH not receiving corticosteroid therapy (*n* = 1)	Detection of characteristic pathogens in bronchoalveolar lavage fluid		*Pneumocystis jirovecii↑*
Lahmer et al. [48]	Critically ill patients with LC (*n* = 120), with FC (*n* = 69) or without FC (*n* = 51); controls (*n* = 120), with FC (*n* = 31) or without FC (*n* = 89)	Retrospective mortality analysis of a prospectively maintained database of 120 patients with cirrhosis, with and without FC, compared with 120 noncirrhotic controls matched by APACHE II score	*Candida↑* *Aspergillus↑*	*C. albicans↑* *C. glabrata↑* *C. krusei↑* *C. tropicalis↑* *C. parapsilosis↑* *C. kefyr↑* *C. guilliermondii↑* *C. nivariensis↑* *Asp. fumigatus↑* *Galactomyces cand↑* *Sacch cerevisiae↑* *Trichosporon asahii↑*
Knooihuizen et al. [47]	HC (*n* = 23); patients with cirrhosis (*n* = 21)	Assessment of the ability of patient neutrophils to kill C. albicans		*C. albicans↑*
Otero Sanchez et al. [71]	Patients with severe AH (*n* = 139); patients with decompensated alcohol‐associated cirrhosis (*n* = 68)		*Aspergillus↑* *Candida↑*	*Pneumocystis jirovecii↑*

The intestinal virome is less well studied but increasingly relevant. Patients with AH show altered viral diversity and expansion of virulent bacteriophages, with changes in *Lactococcus*, *Bacteroides*, *Lactobacillus*, *Escherichia*, *Enterobacter*, *Enterococcus* phages, and herpesviruses reported in association with disease severity [34, 90]. Bacteriophages may worsen or improve ALD depending on whether they destabilize protective bacterial communities or selectively remove pathogenic bacteria [24, 28, 91]. Phage therapy targeting cytolytic *Enterococcus faecalis* reduced cytolysin and attenuated ethanol‐induced liver disease in humanized mice, supporting further clinical evaluation of precision microbiome editing [24, 92]. Clinical translation will require phage–bacteria–host networks to be modified without causing ecological instability or resistance [93, 94].

#### 3.7.3 Microbial Metabolites and Inflammasome Signaling

Microbial products influence pyroptosis through pattern‐recognition receptors, cytosolic sensors, and metabolic regulators. LPS activates TLR4–MyD88–NF‐κB signaling to induce pro‐IL‐1β and NLRP3 expression, and cytosolic LPS can directly activate Caspase‐4/5/11‐dependent noncanonical pyroptosis [58, 59, 95, 96]. Bacterial DNA can activate TLR9 and promote IL‐1β production, while extracellular ATP and potassium efflux provide additional signals for inflammasome assembly [97, 98, 99, 100]. *Candida albicans* activates NLRP3‐mediated macrophage pyroptosis, and candidalysin facilitates inflammasome activation and macrophage escape, linking fungal virulence to lytic inflammatory cell death [15, 101, 102, 103, 104].

By contrast, protective microbial metabolites may restrain inflammasome activation. Butyrate and other short‐chain fatty acids strengthen tight junctions, reduce endotoxemia, support regulatory immunity, and have been shown to attenuate GSDMD‐mediated pyroptosis in experimental alcoholic fatty liver disease [85, 105, 106]. Bile acids can either protect or injure depending on composition, receptor engagement, concentration, and cellular context; farnesoid X receptor (FXR) and Takeda G protein‐coupled receptor 5 (TGR5) signaling may reduce inflammation and improve barrier function, whereas toxic hydrophobic bile acids can promote hepatocyte stress, macrophage activation, and pyroptosis [107, 108, 109, 110, 111]. Tryptophan metabolites that activate aryl hydrocarbon receptor signaling can enhance IL‐22‐dependent antimicrobial defense and may counter dysbiosis‐driven liver injury [112]. These findings suggest that therapy should restore microbial function rather than simply increase or decrease broad taxonomic groups [51, 82, 113].

The anatomical connection between the intestine and liver makes the gut–liver axis a central driver of ALD progression [2, 14, 84]. Alcohol disrupts tight junction proteins, mucus integrity, antimicrobial peptide production, epithelial mitochondrial function, and mucosal immunity, thereby increasing intestinal permeability [70, 114]. Dysbiosis then enriches potentially pathogenic bacteria, fungi, and viruses while reducing protective taxa that produce short‐chain fatty acids and maintain epithelial homeostasis [22, 26]. Portal delivery of LPS, peptidoglycan, bacterial DNA, β‐glucan, and candidalysin activates hepatic pattern‐recognition receptors and links intestinal ecology directly to liver inflammation [22, 115, 116].

Microbial metabolites add another layer of regulation. Short‐chain fatty acids, especially butyrate, support barrier integrity, regulatory immune responses, and energy metabolism, and their reduction may weaken intestinal defense and promote inflammasome activation [15, 85, 106]. Bile acids are transformed by gut microbes and signal through FXR and TGR5, thereby influencing lipid metabolism, antimicrobial programs, inflammation, and fibrosis [117, 118, 119, 120]. Tryptophan‐derived indoles can activate aryl hydrocarbon receptor signaling and IL‐22‐mediated epithelial protection, suggesting that loss of beneficial metabolites may be as important as gain of harmful microbial products [112, 121, 122]. These metabolic functions make the microbiome a therapeutic target beyond its taxonomic composition [82, 123].

### 3.8 ALD and MASLD

ALD and MASLD have traditionally been classified as distinct etiologic entities, but the current steatotic liver disease nomenclature recognizes substantial biological overlap between alcohol exposure and cardiometabolic dysfunction [124, 125]. MASLD is defined by hepatic steatosis together with at least one cardiometabolic risk factor, whereas metabolic dysfunction and ALD (MetALD) identifies individuals meeting MASLD criteria who consume 140–350 g of alcohol per week for women or 210–420 g per week for men [125]. These diagnostic thresholds are useful for classification but should not be interpreted as sharp biological boundaries because alcohol exposure and metabolic dysfunction interact across a continuum and may jointly influence steatosis, inflammation, fibrosis, and clinical outcomes [124, 125].

#### 3.8.1 Shared Pathogenic Pathways in ALD and MASLD

Despite different initiating exposures, ALD and MASLD converge on an overlapping network of downstream hepatic injury pathways, including disordered lipid metabolism, lipotoxic stress, oxidative stress, mitochondrial and endoplasmic reticulum dysfunction, innate immune activation, regulated cell death, gut–liver axis disruption, and hepatic stellate cell‐driven fibrogenesis [10, 45, 125, 126]. In both diseases, hepatic triglyceride accumulation is an early phenotype, but disease progression is more closely related to the generation of toxic lipid species, organelle stress, and the inability of hepatocytes to maintain metabolic and redox homeostasis than to neutral triglyceride storage alone [10, 126]. Mitochondrial dysfunction represents a central point of convergence because impaired fatty acid oxidation, excessive ROS production, altered mitochondrial dynamics, defective mitophagy, and release of mitochondrial danger signals can couple metabolic injury to inflammation and cell death in both ALD and MASLD [33, 45, 125]. The inflammatory response is also shared, with hepatocyte‐derived DAMPs and gut‐derived microbial products activating Kupffer cells, recruited monocytes, neutrophils, and other immune populations, thereby promoting cytokine production and perpetuating hepatocellular injury [10, 38]. Similarly, apoptosis, necroptosis, pyroptosis, ferroptosis, and dysregulated autophagy can contribute to hepatocyte loss and inflammatory amplification in both conditions, although their relative importance varies according to disease stage, cell type, and the dominant upstream insult [48, 52]. Gut dysbiosis and increased intestinal permeability provide another shared mechanistic axis by increasing portal exposure to microbial products and altering short‐chain fatty acids, bile acids, and other microbial metabolites that regulate hepatic metabolism and immunity [10, 37, 127]. At the host level, genetic susceptibility further contributes to both ALD and MASLD because variants in genes such as PNPLA3, TM6SF2, and HSD17B13 influence susceptibility and disease progression across the steatotic liver disease spectrum [11, 12].

#### 3.8.2 Distinct Mechanisms and Pathways

The main distinction between ALD and MASLD lies in their dominant initiating stressors and in the relative contribution of shared downstream mechanisms [124, 125]. In ALD, ethanol metabolism is the defining upstream insult: oxidation of ethanol to acetaldehyde and induction of cytochrome P450 2E1 alter the hepatic NADH/NAD+ redox state, increase ROS generation, impair mitochondrial function, and promote acetaldehyde–protein and acetaldehyde–DNA adduct formation [38, 45]. Alcohol also disrupts intestinal epithelial integrity and mucosal immunity, increasing microbial translocation and sensitizing hepatic innate immune cells to pathogen‐associated molecular patterns [37, 127]. In advanced ALD, particularly AH, intense neutrophilic inflammation may coexist with systemic immune dysfunction and increased susceptibility to bacterial and fungal infection, creating a disease phenotype that is not typical of uncomplicated MASLD [38, 97]. By contrast, MASLD is primarily initiated by systemic metabolic dysfunction, especially insulin resistance, adipose tissue dysfunction, obesity, and Type 2 diabetes, which increase free fatty acid delivery to the liver and stimulate hepatic de novo lipogenesis [126]. When adipose tissue insulin resistance persists, excessive fatty acid flux, hyperinsulinemia, altered adipokine signaling, and nutrient excess favor the formation of lipotoxic lipid species that trigger endoplasmic reticulum stress, mitochondrial dysfunction, oxidative injury, and inflammatory signaling [10, 126]. The extrahepatic context also differs: cardiometabolic disease is an integral component of MASLD biology, whereas AUD, recurrent alcohol exposure, malnutrition, and alcohol‐related immune dysfunction are especially important modifiers of ALD progression [2, 125]. Nevertheless, liver histology alone often cannot reliably assign an alcohol‐related or metabolic etiology because steatosis, ballooning injury, lobular inflammation, and fibrosis substantially overlap between the two diseases [124, 125].

#### 3.8.3 MetALD

MetALD is therefore best understood as an overlap phenotype rather than a simple intermediate category between MASLD and ALD [124, 125, 128]. Metabolic dysfunction can increase hepatic vulnerability to alcohol by increasing fatty acid substrate delivery, lipotoxic stress, insulin resistance, and baseline inflammatory signaling, while alcohol can further impair mitochondrial oxidative capacity, disturb lipid handling, increase oxidative stress, and worsen intestinal barrier dysfunction [125, 126]. Together, these processes can amplify ROS generation, innate immune activation, hepatocyte death, and profibrogenic signaling [124, 125]. Population‐based data support an interaction rather than a purely additive model, as alcohol exposure has been associated with a dose‐dependent increase in significant fibrosis risk when cardiometabolic risk factors are present [129]. A recent systematic review and meta‐analysis further showed that MetALD represents a clinically relevant proportion of the steatotic liver disease population and is associated with adverse outcomes including all‐cause mortality, cardiovascular events, cancer‐related mortality, and hepatic decompensation [130]. However, current evidence is still dominated by observational cohorts, and estimates are affected by differences in alcohol quantification, metabolic criteria, population characteristics, and the possibility of underreported alcohol consumption [125, 129, 130]. Therefore, the present evidence supports a contributory and potentially synergistic interaction between alcohol and metabolic dysfunction, but the magnitude of this interaction is unlikely to be uniform across all patients with MetALD [125, 129].

#### 3.8.4 Translational Implications and Unresolved Questions

The MetALD framework has important implications for risk stratification because patients with steatotic liver disease should be assessed for both cardiometabolic dysfunction and alcohol exposure rather than being assigned to mutually exclusive metabolic or alcohol‐related categories [124, 125, 126]. Accurate classification requires repeated and quantitative assessment of alcohol consumption, since drinking patterns can change over time and self‐reported intake may lead to misclassification around the MASLD–MetALD–ALD boundaries [129]. Similarly, the presence and severity of obesity, diabetes, dyslipidemia, hypertension, and insulin resistance should be incorporated into prognostic assessment because these factors may modify the hepatic effects of alcohol exposure [125, 129]. From a therapeutic perspective, MetALD requires integrated management that addresses both drivers, including reduction or cessation of alcohol exposure together with weight control and treatment of diabetes, dyslipidemia, and other cardiometabolic risk factors [125, 126]. Mechanism‐based clinical trials should also stratify participants by alcohol exposure and metabolic phenotype because failure to recognize overlap disease may dilute treatment effects and contribute to heterogeneous responses in trials designed for ALD or MASLD alone [124, 125]. Future studies should determine whether molecular signatures derived from genetics, metabolomics, microbiome profiling, inflammatory markers, and cell‐death pathways can identify biologically distinct MetALD subgroups that are more informative than alcohol thresholds alone [124, 125]. Longitudinal cohorts with objective alcohol biomarkers, standardized metabolic phenotyping, and repeated assessment of fibrosis are particularly needed to define dose–response relationships and to clarify whether specific pathogenic pathways dominate at different stages of MetALD [125, 129, 130]. Overall, the emergence of MetALD reinforces the concept that ALD and MASLD are connected components of a broader steatotic liver disease spectrum in which distinct initiating factors converge on shared mechanisms of hepatocellular stress, inflammation, cell death, and fibrosis [124, 125, 126].

## 4 Interaction Between Dysbiosis and Other Pathogenic Mechanisms

Gut microbiota dysbiosis in ALD should be viewed as part of an interconnected gut–liver network rather than an isolated pathological event because alterations in microbial composition, intestinal barrier integrity, microbial translocation, and metabolite signaling can interact with multiple host injury pathways [31, 37, 127]. The following subsections therefore summarize four major dimensions of this bidirectional crosstalk: the influence of microbiota on ethanol metabolism and toxic metabolite exposure; reciprocal links with oxidative stress and redox imbalance; modulation of hepatic inflammatory and immune responses; and regulation of hepatocyte death pathways, including pyroptosis, apoptosis, necroptosis, and ferroptosis [31, 85, 109]. Conversely, alcohol‐induced oxidative injury, inflammation, and hepatocyte death can further impair intestinal barrier function and reshape the gut microenvironment, creating a feed‐forward loop that may amplify ALD progression [31, 37].

### 4.1 Microbiota and Alcohol Metabolism

Alcohol metabolism is the initiating step in the onset and progression of ALD (Figure 1). First, gut microbiota directly participate in the metabolism of alcohol. Certain gut bacteria (such as some strains of Escherichia coli and Klebsiella pneumoniae) express alcohol dehydrogenase and cytochrome P450 2E1 (CYP2E1), which oxidize ethanol to acetaldehyde [131]. Although this reduces the amount of ethanol entering the liver via the portal vein, it also leads to the accumulation of acetaldehyde in the intestinal lumen and portal circulation. Second, gut microbiota indirectly regulate hepatic alcohol metabolism through their metabolites. Gut dysbiosis alters the liver NAD^+^/NADH ratio, thereby affecting hepatocyte alcohol dehydrogenase activity [132]. Moreover, microbial metabolites such as short‐chain fatty acids act as signaling molecules via the gut‐liver axis to influence the expression of genes involved in fatty acid oxidation and de novo lipogenesis, ultimately alleviating alcohol‐induced hepatic lipid metabolic disorders [127, 133].

**FIGURE 1:**
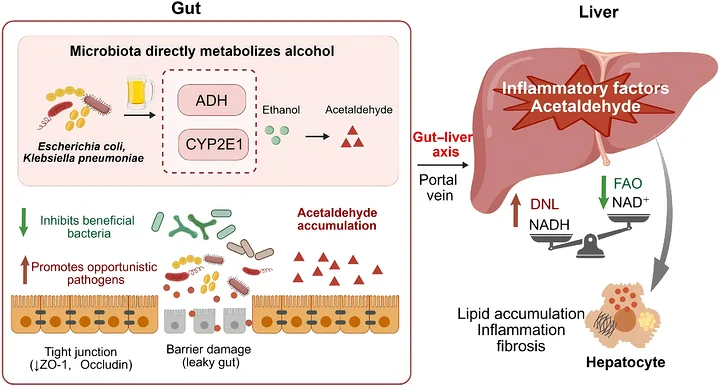
Interaction between gut microbiota and alcohol metabolism in ALD. Chronic alcohol intake depletes beneficial commensal bacteria while promoting opportunistic pathogens (e.g., *Escherichia coli* and *Klebsiella pneumoniae*) in the gut. Some bacterial species directly metabolize ethanol to acetaldehyde via ADH and CYP2E1. This process reduces the amount of ethanol entering the liver via the portal vein but leads to acetaldehyde accumulation in the intestinal lumen and portal circulation. Acetaldehyde directly damages intestinal epithelial cells, disrupts tight junction proteins (ZO‐1 and occludin), and impairs the intestinal barrier (“leaky gut”), which promotes the proliferation of opportunistic pathogens and inhibits beneficial bacteria. Through the gut–liver axis, ethanol, acetaldehyde, and microbial metabolites such as SCFAs reach the liver via the portal vein. Gut dysbiosis alters the hepatic NAD^+^/NADH ratio, affecting hepatocyte ADH activity and influencing FAO and DNL. Together, the resulting lipid accumulation, inflammation, and fibrosis drive ALD progression. Abbreviations: ADH, alcohol dehydrogenase; CYP2E1, cytochrome P450 2E1; DNL, de novo lipogenesis; FAO, fatty acid oxidation; NAD^+^, oxidized nicotinamide adenine dinucleotide; NADH, reduced nicotinamide adenine dinucleotide; ZO‐1, zonula occludens‐1.

Alcohol metabolites also shape the structure and function of the gut microbiota. Acetaldehyde, a highly reactive toxic metabolite, directly damages intestinal epithelial cells, disrupts the intestinal barrier, and alters the intestinal microenvironment, thereby promoting the proliferation of opportunistic pathogens and inhibiting the growth of beneficial bacteria [134]. In summary, there is a bidirectional interaction between gut microbiota dysbiosis and alcohol metabolism. Alcohol consumption alters the microbial composition and in turn, microbial changes influence ethanol metabolism efficiency and the accumulation of toxic intermediates, further exacerbating alcohol‐induced liver injury. Therefore, targeting the gut microbiota to modulate alcohol metabolism pathways may represent a novel strategy to alleviate liver injury in ALD [135].

### 4.2 Microbiota and Oxidative Stress

Alcohol‐induced gut microbiota dysbiosis is an important driver that exacerbates hepatic oxidative stress (Figure 2). On one hand, dysbiosis aggravates oxidative stress [135, 136]. LPS, a cell wall component of dysbiotic bacteria (such as Enterobacteriaceae), translocates to the liver through the impaired intestinal barrier. LPS activates the TLR4 signaling pathway in hepatic Kupffer cells, triggering the downstream NADPH oxidase complex to generate large amounts of ROS directly attack hepatocyte lipids, proteins, and DNA, leading to oxidative damage [137]. On the other hand, certain beneficial bacteria and their metabolites possess antioxidant effects. For example, short‐chain fatty acid‐producing bacteria (such as *Roseburia* and *Faecalibacterium*) enhance the antioxidant defense capacity of intestinal epithelial cells, reducing ROS production and release [138, 139, 140].

**FIGURE 2:**
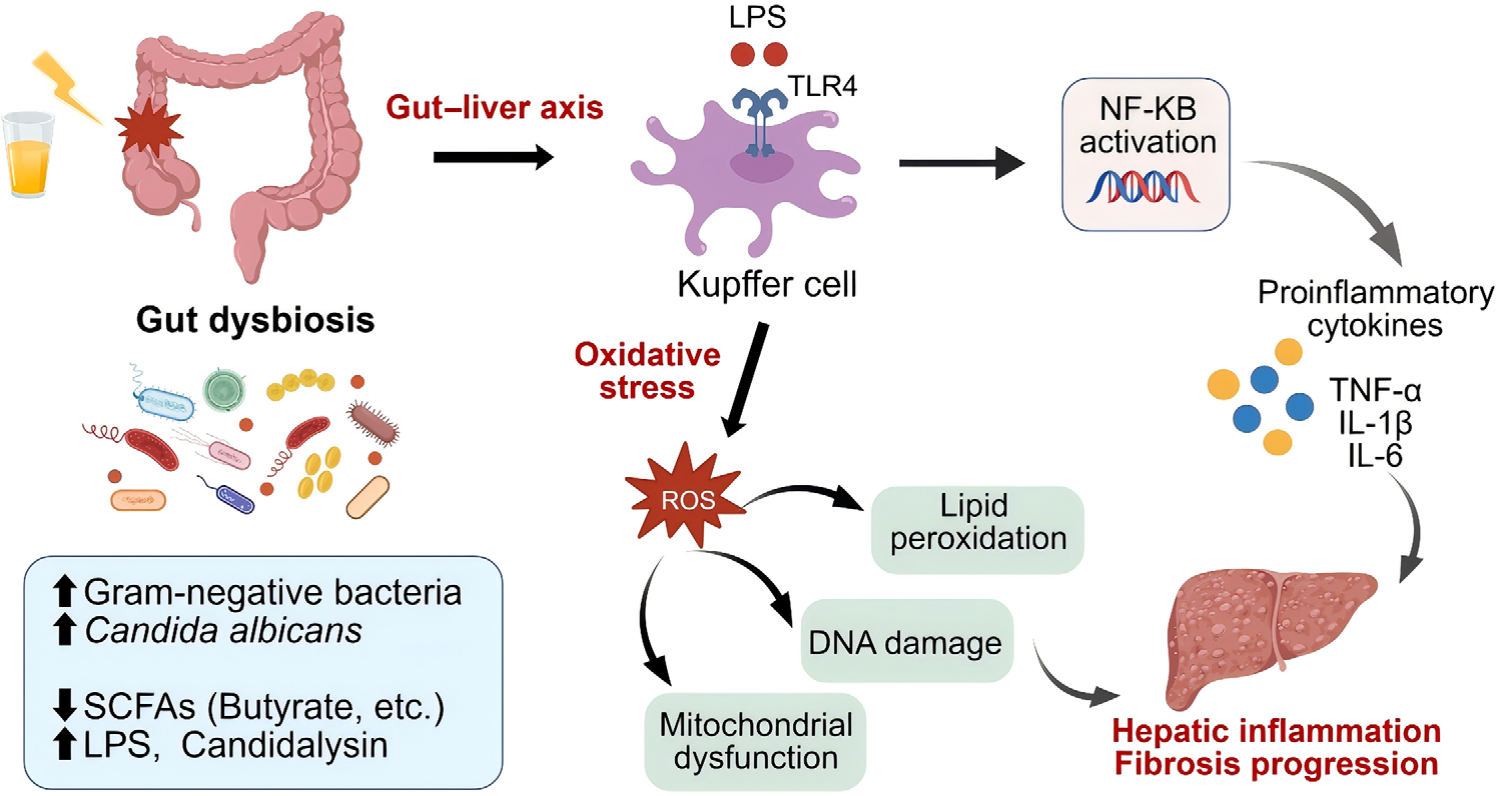
Gut dysbiosis promotes hepatic oxidative stress and inflammation through the gut–liver axis in ALD. Chronic alcohol exposure induces intestinal dysbiosis, characterized by the expansion of Gram‐negative bacteria and *Candida albicans*, reduced concentrations of beneficial SCFAs such as butyrate, and increased translocation of microbial products including LPS and candidalysin. These bacterial and fungal components reach the liver via the gut–liver axis, where LPS activates TLR4 on Kupffer cells, leading to NF‐κB activation and the release of proinflammatory cytokines, including TNF‐α, IL‐1β, and IL‐6. Simultaneously, activated Kupffer cells generate oxidative stress and overproduce ROS, which in turn cause lipid peroxidation, DNA damage, and mitochondrial dysfunction. Together, inflammatory signaling and oxidative injury accelerate hepatic inflammation and fibrosis progression. Abbreviations: IL, interleukin; LPS, lipopolysaccharide; NF‐κB, nuclear factor kappa‐B; ROS, reactive oxygen species; SCFAs, short‐chain fatty acids; TLR4, Toll‐like receptor 4; TNF‐α, tumor necrosis factor‐alpha.

Oxidative stress also reshapes the composition of the gut microbiota in a reciprocal manner. Excessive ROS disrupt tight junction proteins (such as ZO‐1 and occludin) between intestinal epithelial cells, exacerbating intestinal permeability and bacterial translocation [141]. Meanwhile, changes in the intestinal redox environment (such as increased oxidation potential and nitrite accumulation) selectively promote the proliferation of facultative anaerobic pathogens (such as Enterobacteriaceae) while inhibiting oxygen‐sensitive strict anaerobic commensals, further worsening dysbiosis [142]. In addition, oxidative stress‐mediated bile acid metabolism disorders alter the supply of nutrients to the gut microbiota, indirectly affecting microbial composition [143]. Therefore, a healthy gut microbiota is an important factor in maintaining hepatic redox homeostasis. Targeting the gut microbiota to restore intestinal redox balance represents a potential strategy for intervening in ALD.

### 4.3 Microbiota and Inflammatory Immunity

Inflammatory immune activation is a key link in the progression of ALD from simple steatosis to hepatitis and liver fibrosis (Figure 2). The gut microbiota plays an important regulatory role in this process [144, 145]. Alcohol‐induced gut dysbiosis leads to an increase in Gram‐negative bacteria in the intestine, with massive release of their cell wall component LPS. After entering the liver through the portal vein, LPS binds to the TLR4 receptor on the surface of Kupffer cells, activates the NF‐κB signaling pathway, and promotes the production of proinflammatory cytokines such as tumor necrosis factor‐α, IL‐1β, and IL‐6 [74, 146]. In addition, alterations in the gut fungal community participate in the inflammatory response [137]. Candidalysin, produced by *Candida albicans*, directly damages hepatocytes and activates the inflammasome, further exacerbating liver inflammation [85]. Gut dysbiosis also weakens intestinal immune barrier function by reducing the production of anti‐inflammatory metabolites such as short‐chain fatty acids [147, 148].

The inflammatory immune response also acts back on the gut microbiota. Alcohol‐induced liver inflammation promotes the entry of proinflammatory cytokines into the circulatory system, affecting the intestinal immune environment and altering the composition and function of the gut microbiota [149]. At the same time, increased intestinal permeability under inflammatory conditions allows more bacterial products to enter the portal vein [150]. Studies have shown that modulating the gut microbiota (such as supplementing with butyrate‐producing bacteria or fecal microbiota transplantation) alleviates alcohol‐induced hepatic inflammatory responses and reduces serum proinflammatory cytokine levels [151]. Therefore, targeting the gut microbiota to restore intestinal immune homeostasis is an important direction for controlling the progression of ALD [152, 153].

### 4.4 Microbiota and Cell Death

Cell death is the direct executioner of liver injury in ALD, involving multiple forms of programmed cell death such as pyroptosis, apoptosis, and necroptosis [48, 50, 51] (Figure 3). The gut microbiota exerts important effects on liver cell death processes through its metabolites and structural components. LPS is a key microbial signal regulating cell death [154, 155, 156]. After entering the liver, LPS activates the NLRP3 inflammasome in Kupffer cells, inducing Caspase‐1 activation and GSDMD‐mediated pyroptosis and triggers hepatocyte apoptosis and necroptosis through the TLR4 signaling pathway [157, 158, 159]. Furthermore, candidalysin derived from gut fungi directly damages hepatocyte membranes and promotes inflammasome‐dependent cell death [85]. In contrast, beneficial microbial metabolites such as butyrate inhibit GSDMD‐mediated pyroptosis and alleviate alcohol‐induced hepatocyte injury [109].

**FIGURE 3:**
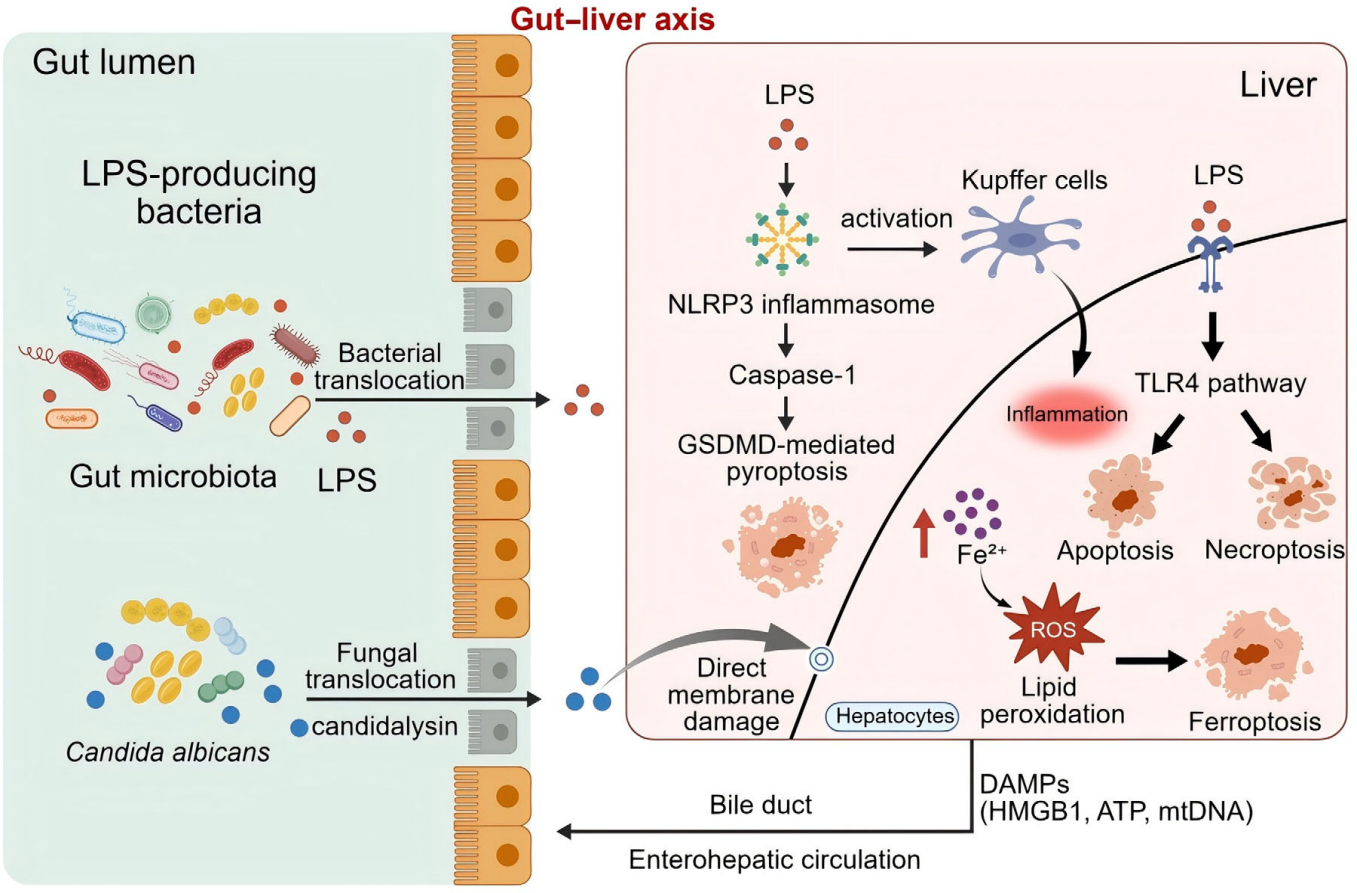
Interaction between gut microbiota and cell death in ALD. In the gut, alcohol‐induced dysbiosis promotes the expansion of LPS‐producing bacteria and Candida albicans, accompanied by increased intestinal permeability that facilitates bacterial and fungal translocation. Translocated LPS reaches the liver and activates the NLRP3 inflammasome in Kupffer cells, leading to Caspase‐1 activation and GSDMD‐mediated pyroptosis. Concurrently, LPS activates the TLR4 pathway, triggering hepatic inflammation and promoting apoptosis and necroptosis. C. albicans‐derived candidalysin directly damages hepatocyte membranes. Additionally, iron overload (Fe^2^^+^) drives ROS production and lipid peroxidation, culminating in ferroptosis. Dying hepatocytes release DAMPs including HMGB1, ATP, and mtDNA into the enterohepatic circulation, which are transported back to the gut via bile ducts, perpetuating a self‐reinforcing cycle of gut barrier injury and hepatic cell death. Abbreviations: ATP, adenosine triphosphate; DAMPs, damage‐associated molecular patterns; Fe^2^^+^, ferrous iron; GSDMD, gasdermin D; HMGB1, high‐mobility group box 1; LPS, lipopolysaccharide; mtDNA, mitochondrial DNA; NLRP3, NOD‐like receptor family pyrin domain containing 3; ROS, reactive oxygen species; TLR4, Toll‐like receptor 4.

Hepatocyte death reshapes the composition of the gut microbiota and exacerbates microbial translocation in a reciprocal manner. DAMPs released from apoptotic and necrotic hepatocytes (such as high mobility group 1 [HMGB1], ATP, and mitochondrial DNA) activate pattern recognition receptors in the liver and local intestinal tissues, induce local inflammatory responses, and subsequently disrupt the intestinal epithelial barrier, thereby exacerbating gut dysbiosis [160, 161]. Importantly, the loss of cell membrane integrity resulting from necroptosis and pyroptosis releases large amounts of intracellular contents into the extracellular space, and when these DAMPs are excreted into the intestine via the biliary tract, they alter the intestinal immune microenvironment and affect microbial colonization selection [52]. In addition, ferroptosis, a form of cell death that has gained considerable attention in recent years, is characterized by the accumulation of iron ions and lipid peroxidation products, which not only directly damage hepatocytes but also affect intestinal redox status through enterohepatic circulation [44, 53]. Studies have shown that inhibiting ferroptosis and modulating the gut microbiota alleviate ALD [54]. Therefore, an in‐depth understanding of the regulatory network between the gut microbiota and cell death may provide new therapeutic targets for ALD.

Together, these findings collectively indicate that gut microbiota interacts with various major pathogenic mechanisms, thereby promoting the progression of ALD (Figure 4).

**FIGURE 4:**
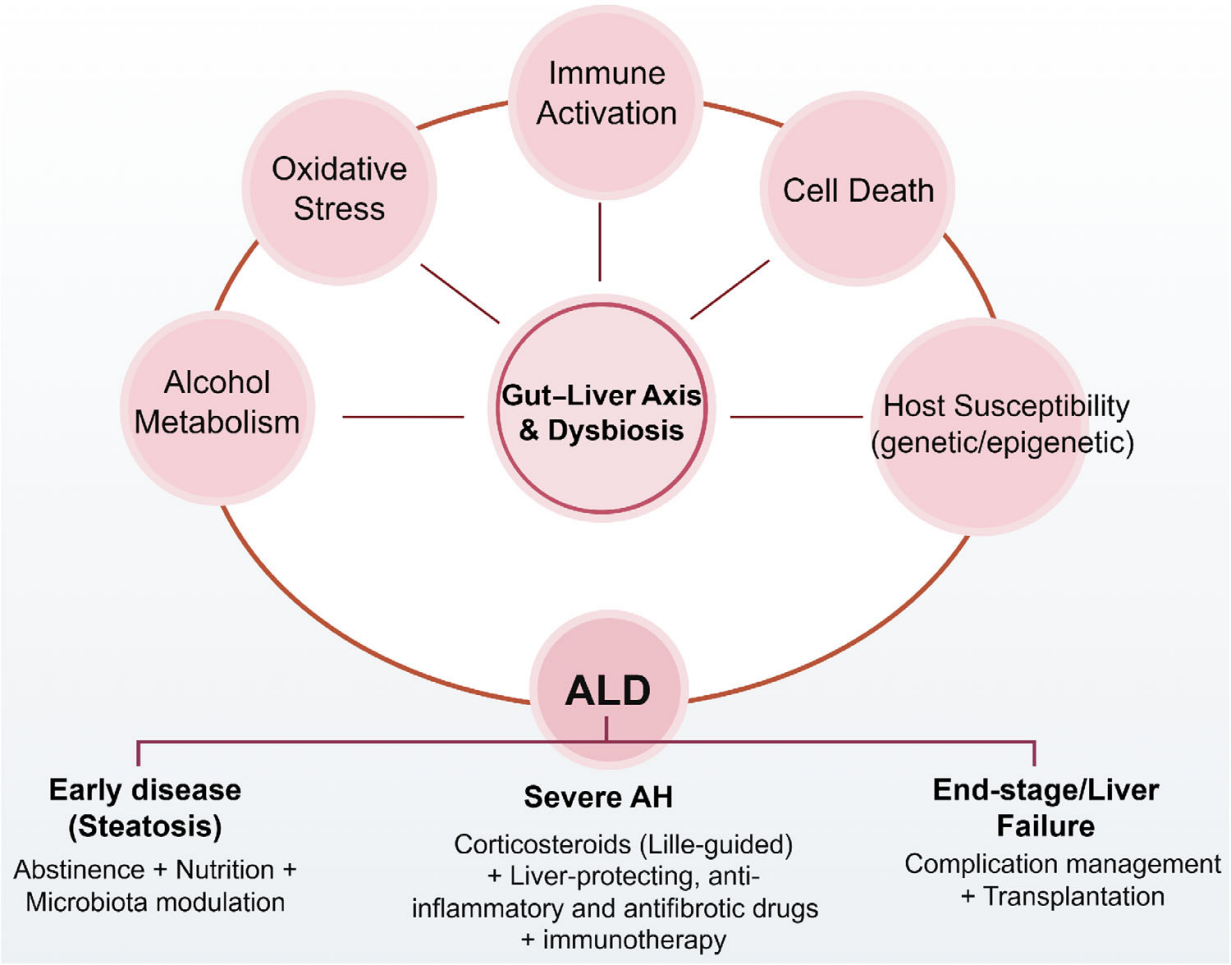
Integrated pathogenesis and therapeutic landscape of ALD. ALD is characterized by disruption of the gut–liver axis and microbial dysbiosis, which interact with abnormal alcohol metabolism, oxidative stress, immune activation, regulated cell death, and host genetic and epigenetic susceptibility. Disease severity ranges from early steatosis to severe alcohol‐associated hepatitis (AH) and end‐stage liver failure. Treatment should therefore be tailored to disease stage and the dominant pathogenic mechanisms. Abbreviations: ALD, alcohol‐associated liver disease; AH, alcohol‐associated hepatitis.

## 5 Treatment

Current management of ALD includes sustained abstinence, treatment of AUD, nutritional support, infection screening, and management of cirrhosis‐related complications [25, 42, 53, 162]. For severe AH, prednisolone should be restricted to carefully selected patients, and early discontinuation in nonresponders should be guided by the Lille score [42, 86, 163]. N‐acetylcysteine (NAC) combined with prednisolone improved short‐term survival in one randomized trial but did not improve longer‐term mortality [69]. Early liver transplantation improves survival in highly selected patients with severe AH who fail medical therapy, but it requires multidisciplinary assessment of relapse risk, psychosocial support, and equitable access [52, 164, 165]. However, these established treatment options remain largely supportive or stage‐restricted, and precise mechanism‐based therapies that can be broadly applied are still lacking [165]. Given that alcohol exposure, metabolic stress, gut–liver axis dysfunction, immune dysregulation, and regulated cell death jointly drive disease progression, emerging therapeutic strategies targeting these key pathogenic mechanisms warrant further investigation. In this section, we summarize clinical and preclinical therapeutic approaches that target the core pathways of ALD, with an emphasis on strategies that have entered clinical testing or shown promising preclinical evidence [1, 14].

### 5.1 Therapies Targeting Ethanol Metabolism, Oxidative Stress, and Metabolic Injury

Ethanol metabolism promotes acetaldehyde accumulation, CYP2E1‐dependent ROS generation, mitochondrial dysfunction, glutathione depletion, lipid peroxidation, and hepatocellular sensitization to inflammatory injury [39]. Antioxidant and metabolic therapies are therefore rational approaches, but their benefits depend on disease stage and on whether oxidative injury is the dominant driver rather than only one component of advanced inflammatory liver failure [31, 39, 166]. The best clinical evidence in this category is NAC added to prednisolone, which improved 1‐month survival in severe AH but did not significantly improve 6‐month survival [69]. Metadoxine has been reported to improve 3‐ and 6‐month survival in severe AH, but the evidence base remains smaller than that for corticosteroids and requires further confirmation in larger multicenter trials [42, 45]. Experimental antioxidant and antiferroptotic strategies, including agents that reduce lipid peroxidation and oxidative stress‐mediated ferroptosis, have attenuated alcohol‐induced liver injury in animal models [166, 167]. Overall, therapies targeting oxidative stress and metabolic injury should currently be considered adjunctive or early‐stage interventions unless future trials demonstrate durable survival benefit in severe AH [39, 69].

### 5.2 Therapies Targeting Innate Immunity and Cytokine Signaling

Alcohol‐induced liver injury activates innate immunity through both DAMPs released from injured hepatocytes and pathogen‐associated molecular patterns translocated from the gut [41, 95]. This dual mechanism explains why anti‐inflammatory therapy remains clinically important but also why broad immune suppression may be dangerous in patients who already have infection susceptibility and immune paralysis [43, 86]. The STOPAH trial showed that prednisolone produced a limited short‐term benefit and did not improve 90‐day or 1‐year mortality, whereas pentoxifylline did not improve survival [86]. The Lille score is therefore essential because it identifies corticosteroid nonresponders early and limits unnecessary steroid exposure [163]. IL‐1 pathway inhibition has produced mixed results: anakinra‐based strategies did not clearly outperform prednisone, and canakinumab has not shown sufficient benefit for routine use in severe AH [168, 169]. In contrast to broad inflammatory blockade, the IL‐22 agonist F‐652 was well tolerated in a Phase 1/2 study and was associated with improvements in MELD score, Lille score, inflammatory markers, and regeneration markers [7]. Granulocyte colony‐stimulating factor (G‐CSF) has shown survival signals in meta‐analyses of randomized trials, but heterogeneity between studies and regions prevents universal recommendation [170]. These data support selective, biomarker‐guided immune modulation rather than indiscriminate suppression of inflammatory pathways in severe AH [39, 169].

### 5.3 Therapies Targeting Regulated Cell Death and Inflammasome–Pyroptosis Pathways

Regulated cell death is an important therapeutic target because hepatocyte apoptosis, necroptosis, ferroptosis, and pyroptosis convert metabolic stress into inflammation, fibrosis, and regenerative demand [19, 59, 65, 93]. Among these processes, gasdermin‐mediated pyroptosis is particularly relevant because it links microbial signals, oxidative stress, inflammasome activation, cytokine release, and membrane rupture [56, 75, 90, 102]. The Caspase‐11/4–GSDMD pathway is activated in experimental models and in patients with AH, supporting the translational relevance of pyroptosis in ALD [90]. TXNIP overexpression, hepatocyte mitochondrial dysfunction, METTL3‐mediated Kupffer cell pyroptosis, and NLRP3 activation have also been implicated in alcohol‐induced pyroptotic injury [73, 94, 102]. Preclinical studies show that inhibition of NLRP3‐related signaling, GSDMD activation, oxidative stress, or upstream danger signals can reduce alcohol‐induced liver injury [102, 117, 171]. Butyrate, hydrogen gas, capsaicin, proanthocyanidins, and other agents have been reported to attenuate ethanol‐induced injury partly by inhibiting inflammasome or pyroptosis pathways in experimental systems [171]. Despite these promising data, clinical translation remains limited because pyroptosis and inflammasome signaling also contribute to antimicrobial defense in patients with severe AH [78, 172]. Future antipyroptosis therapy should therefore be short‐course, stage‐specific, and biomarker‐guided rather than broad, prolonged blockade of host‐defense pathways [78, 102, 105].

### 5.4 Therapies Targeting the Gut–Liver Axis and Microbiota Dysbiosis

The gut–liver axis is a major therapeutic target because alcohol disrupts the intestinal barrier, induces bacterial and fungal dysbiosis, alters microbial metabolites, and increases portal delivery of inflammatory ligands [107, 145, 168]. Probiotics, prebiotics, synbiotics, postbiotics, and dietary strategies aim to restore microbial functions such as short‐chain fatty acid production, epithelial barrier protection, bile acid transformation, and immune regulation [134]. Clinical studies of probiotics have reported improvements in liver enzymes, endotoxemia, cytokines, or drinking‐related assessments, although strain specificity, sample size, and disease‐stage heterogeneity limit routine recommendations [57]. FMT provides broader restoration of the microbial ecosystem and has shown encouraging signals for feasibility, survival, infection reduction, and microbiome remodeling in pilot, open‐label, randomized, and meta‐analytic studies of severe AH or alcohol‐associated acute‐on‐chronic liver failure [66]. Precision phage therapy is mechanistically attractive because cytolysin‐positive *Enterococcus faecalis* is associated with severe AH and targeted phages reduced liver cytolysin and ethanol‐induced liver injury in humanized mice [88]. Antifungal or anticandidalysin strategies are also plausible because intestinal fungi and candidalysin‐producing *Candida albicans* can promote alcohol‐associated liver injury in experimental and translational studies [116, 138]. Rifaximin may reduce bacterial overgrowth and translocation, and a pilot study in severe AH reported fewer infections and clinical complications when it was added to standard therapy [55, 61]. Future microbiota‐directed trials should integrate bacterial, fungal, viral, metabolomic, bile acid, permeability, and inflammatory readouts so that treatment is matched to microbial function rather than to taxonomy alone [107, 163, 170].

### 5.5 Therapies Targeting Bile Acid Signaling, Metabolic Repair, and Regeneration

Bile acid signaling connects gut microbial metabolism with intestinal barrier function, hepatic lipid metabolism, inflammation, and regeneration, making it a mechanism that overlaps with both microbiota‐directed and metabolic therapy [131, 173, 174]. In experimental ALD, FXR and TGR5 agonists reduced steatosis, inflammation, and liver injury, while modulation of the intestinal FXR–FGF15 axis improved ethanol‐associated liver injury in mice. IL‐22‐based therapy also supports this repair‐oriented approach because engineered intestinal bacteria producing IL‐22 reduced ethanol‐induced liver disease in mice and recombinant F‐652 showed clinical tolerability and biomarker improvement in AH. Larsucosterol (DUR‐928), an endogenous sulfated oxysterol that epigenetically regulates inflammatory, lipid‐metabolic, and cell‐stress pathways, has demonstrated a favorable safety profile and preliminary efficacy signals in early‐phase trials; although the Phase 2b AHFIRM trial did not meet its primary composite endpoint, prespecified analyses showed significant reductions in 90‐day mortality in the US subgroup. G‐CSF is another regenerative strategy with reported survival signals in randomized‐trial meta‐analysis, although heterogeneity and inconsistent external validation require cautious interpretation [172]. Because repair, metabolism, and inflammation are intertwined in ALD, these strategies may ultimately be most useful in combination with abstinence care, nutrition, infection surveillance, and mechanism‐enriched patient selection [78, 105, 175].

### 5.6 Stage‐Specific and Mechanism‐Guided Therapeutic Framework

In early steatosis or compensated disease, the main management priorities are alcohol abstinence, AUD treatment, nutritional optimization, metabolic risk modification, and prevention of progression [1, 78]. In severe AH, treatment decisions should combine corticosteroid eligibility, infection screening, early Lille‐score reassessment, renal function, nutritional status, and transplant evaluation [20, 113, 164]. In patients with dysbiosis‐dominant phenotypes, microbiota‐directed interventions such as probiotics, FMT, phage therapy, rifaximin, or antifungal approaches should be evaluated using microbial signatures and predefined safety criteria [116]. In patients with inflammasome or pyroptosis‐enriched signatures, pyroptosis‐targeted approaches should be evaluated with biomarkers such as GSDMD fragments, IL‐18, IL‐1β, microbial translocation markers, and inflammasome‐related transcriptomic patterns [102, 116]. In advanced liver failure or steroid‐nonresponsive severe AH, early liver transplantation remains the most effective rescue therapy for carefully selected candidates [164]. Future ALD trials should therefore move from broad diagnostic enrollment toward mechanism‐enriched designs with endpoints that include mortality, transplant‐free survival, infection, renal failure, alcohol relapse, quality of life, and target engagement [164]. Table 3 summarizes representative preclinical and clinical treatment strategies for ALD.

**TABLE 3:** Established, clinical‐stage, and investigational therapies for alcohol‐associated liver disease and alcohol‐associated hepatitis.

Therapy	Mechanism/target	Stage	Representative trial(s)/NCT	Status and key results
**A. Established/guideline‐recommended**				
Corticosteroids (prednisolone)	Broad anti‐inflammatory	Standard of care (severe AH)	STOPAH (ISRCTN88782125)	28‐day survival benefit (OR 0.72); no 90‐day or 1‐year benefit; serious infections 13 vs. 7%; ∼40% nonresponse (Lille ≥0.45 → discontinue)
Early liver transplantation	Definitive replacement therapy	Standard of care (selected nonresponders)	Mathurin 2011; Lee 2018; Louvet 2022	Markedly improved survival in carefully selected patients; limited by organ supply and selection policies
AUD pharmacotherapy (baclofen, acamprosate, naltrexone)	Relapse prevention	Standard of care (adjunct)	ACG/AASLD guidelines	Safe in advanced liver disease; prescribed to <2% of patients with ALD
Nutritional support	Corrects protein‐energy malnutrition	Standard of care (adjunct)	ACG/AASLD guidelines	Recommended for all patients with AH
**B. Positive Phase 2 results/advanced development**				
Larsucosterol (DUR‐928)	Epigenetic regulator (DNMT inhibitor; sulfated oxysterol)	Phase 3 (initiated 2026)	NCT03432260 (Phase 2a); NCT04563026 (AHFIRM Phase 2b)	Phase 2b trial did not meet the primary endpoint; 90‐day mortality −41/−35% overall, −57/−58% in US subgroup; US FDA Fast Track and Breakthrough Therapy designations
F‐652 (eflepedocokin alfa)	IL‐22 agonist; hepatocyte regeneration	Phase 2	NCT02655510; AlcHepNet/ITAALD	Safe; Day‐7 Lille response in 83%; ongoing NIAAA trial evaluating concurrent treatment of AH and AUD
N‐acetylcysteine + prednisolone	Glutathione replenishment; antioxidant	Phase 3 (NACAH, ongoing)	NCT00863785; NCT03069300	Improved 1‐month survival (8 vs. 24% mortality) but not 6‐month survival; Phase 3 trial recruiting
Metadoxine	Accelerates acetaldehyde clearance; antioxidant	Phase 2	NCT02161653	Improved 3‐ and 6‐month survival in a single‐center, open‐label study; confirmation is needed
Fecal microbiota transplantation	Restores gut microbial ecosystem	Phase 2	Pande 2023 (RCT); NCT05006430; NCT05285592	90‐day survival of 75 vs. 56.6% with prednisolone; fewer infection deaths; capsule formulations in testing
**C. Mixed or conflicting evidence**				
G‐CSF	Bone‐marrow mobilization; regeneration	Phase 2	NCT01820208; GRAFT study; GPreAH; NCT06890039 (with TAK‐242)	Benefit in Indian RCTs and meta‐analysis (90‐day mortality OR 0.28); negative in European ACLF; regional heterogeneity
Rifaximin	Nonabsorbable antibiotic	Phase 2	RIFA‐AH pilot (Jiménez 2022)	Fewer infections and ACLF; no mortality benefit
Probiotics (e.g., Lactobacillus GG)	Microbiome modulation	Phase 1/2	NCT05178069; Kirpich 2008; Han 2015	Improved liver enzymes and drinking measures; clinical endpoints pending
**D. Failed, negative, or terminated (lessons)**				
Pentoxifylline	Phosphodiesterase/TNF inhibition	Phase 3 trial negative	STOPAH (ISRCTN88782125)	No survival benefit at any time point
Infliximab	Anti‐TNF	Phase 2 trial stopped	Naveau 2004	Excess infections and deaths with prednisolone
Etanercept	Anti‐TNF	Phase 2 trial negative	Boetticher 2008	Higher 6‐month mortality
Anakinra‐based regimens	IL‐1 receptor blockade	Phase 2; negative/stopped	Szabo 2022; NCT04072822	No benefit; inferior to prednisone (stopped early)
Canakinumab	Anti‐IL‐1β	Phase 2; primary endpoint not met	Vergis 2025	Safe; histological improvement trend only
Selonsertib	ASK1 inhibitor	Phase 2 trial negative	NCT02854631	No added benefit over prednisolone
Emricasan	Pan‐caspase inhibitor	Terminated	NCT01912404	Stopped for safety concerns
Obeticholic acid	FXR agonist	Terminated	NCT02039219	Stopped over safety concerns
Amoxicillin–clavulanate + prednisolone	Broad‐spectrum antibiotic	Phase 3 trial negative	AntibioCor (Louvet 2023)	No survival benefit
**E. Early clinical/exploratory (Phase 1–2)**				
INT‐787	Next‐generation FXR agonist	Phase 2a	NCT05639543 (FRESH)	Recruiting
TAK‐242	TLR4 inhibitor	Phase 2	NCT04620148; NCT06890039 (with G‐CSF)	Recruiting
Plasma exchange	Removal of inflammatory mediators	Phase 2	NCT06355726	Recruiting
Bovine colostrum/IMM‐124E	Gut barrier protection; anti‐LPS	Phase 2/3	NCT02473341; NCT01968382	Ongoing
Digoxin (repurposed)	Immunomodulation	Phase 2	NCT05014087	Recruiting
Hyaluronic acid 35 (HA35)	Gut barrier protection	Phase 2	NCT05018481	Recruiting
Omega‐5 fatty acids	PPARγ agonism; antioxidant	Phase 2	NCT03723586	Ongoing
Extended low‐dose prednisolone	Prolonged immunomodulation	Phase 2	NCT06155760	Recruiting
Intensive nutritional intervention	Malnutrition reversal	Phase 2/3	NCT06131177	Recruiting (target *n* = 2000)

Abbreviations: AH = alcohol‐associated hepatitis; ALD = alcohol‐associated liver disease; AUD = alcohol use disorder; NCT = ClinicalTrials.gov identifier; OR = odds ratio; HR = hazard ratio; MELD = Model for End‐Stage Liver Disease; FMT = fecal microbiota transplantation; G‐CSF = granulocyte colony‐stimulating factor; FXR = farnesoid X receptor; TNF = tumor necrosis factor; IL = interleukin; NAC = N‐acetylcysteine; ACLF = acute‐on‐chronic liver failure. Trial phases and statuses verified against ClinicalTrials.gov and published sources as of August 2026.

### 5.7 Clinical‐Stage Drugs and Future Directions

Despite decades of investigation, no pharmacotherapy has been approved for AH, and the therapeutic landscape remains defined by a single modestly effective agent (corticosteroids) and a life‐saving but rationed procedure (early liver transplantation) [176]. Over the past decade, however, mechanistic advances have translated into a growing number of clinical trials. This section reviews agents that have reached clinical testing (Table 3), extracts the lessons of negative and failed trials, and proposes priorities for the next generation of studies [177].

#### 5.7.1 Agents in Clinical Development

*Epigenetic and Pleiotropic Modulators*. Larsucosterol (DUR‐928; 25‐hydroxycholesterol‐3‐sulfate) is an endogenous sulfated oxysterol that inhibits DNA methyltransferases (DNMT1, DNMT3a, and DNMT3b), thereby concurrently modulating inflammatory signaling, lipid metabolism, and cell‐death pathways. In a Phase 2a open‐label study (NCT03432260; *n* = 19), all treated patients survived the 28‐day study period, 89% achieved a Day‐7 Lille score <0.45, and no drug‐related serious adverse events occurred [178]. The subsequent Phase 2b AHFIRM trial (NCT04563026; *n* = 307) did not meet its primary composite endpoint of 90‐day mortality or liver transplantation; larsucosterol nevertheless reduced 90‐day mortality by 41% (30 mg; *p* = 0.068) and 35% (90 mg; *p* = 0.124) versus placebo overall, and by 57% (*p* = 0.014) and 58% (*p* = 0.008), respectively, in the prespecified US subgroup, which comprised approximately 76% of the study population [179]. The drug has received United States Food and Drug Administration Fast Track and Breakthrough Therapy designations, and a Phase 3 registration trial powered for 90‐day mortality was initiated in early 2026, with topline results expected around 2028. Larsucosterol is among the most advanced pharmacotherapies in the AH development pipeline.

*Liver Regeneration*. F‐652 (eflepedocokin alfa) is a recombinant human IL‐22/IgG2‐Fc fusion protein that promotes hepatocyte proliferation and exerts antioxidant and antiapoptotic effects without systemic immunosuppression because IL‐22 receptors are largely restricted to epithelial cells. In a Phase 2a dose‐escalation study (NCT02655510; *n* = 18; MELD 11–28), F‐652 was safe, achieved Day‐7 Lille responses in 83% of patients, reduced MELD scores and inflammatory markers, and was associated with 11.1% 90‐day mortality versus 27% in matched historical controls [180]. Building on these results, the NIAAA‐funded AlcHepNet (ITAALD) network has launched a multicenter, double‐blind Phase 2 trial comparing F‐652 with prednisone in severe AH, factorially crossed with AUD treatment (acamprosate plus motivational interviewing versus usual care), thereby integrating treatment of liver disease with treatment of the underlying AUD [181].

*Hematopoietic Growth Factors*. G‐CSF mobilizes bone‐marrow‐derived progenitors and may enhance hepatic regeneration. Randomized trials in India reported survival benefits (for example, 90‐day mortality of 35.7 vs. 71.4% with placebo; NCT01820208) and the more recent GPreAH trial showed that G‐CSF improved prednisolone responsiveness and 90‐day survival in steroid‐eligible patients [182, 183]. A meta‐analysis of randomized trials found reduced 90‐day mortality overall (OR 0.28); however, the large German GRAFT study in 176 patients with acute‐on‐chronic liver failure showed no benefit, and efficacy appears largely confined to Asian populations, highlighting regional heterogeneity in patient selection, disease phenotype, and standard of care [172, 184]. A Phase 2 trial combining G‐CSF with the Toll‐like receptor 4 inhibitor TAK‐242 (NCT06890039) is now recruiting, reflecting growing interest in rational combination therapy [181].

*Gut–Liver Axis*. Microbiome‐directed therapies have produced promising recent data. In an open‐label randomized trial of 120 patients with severe AH, 7 days of naso‐duodenal fecal microbiota transplantation (FMT) from healthy donors improved 90‐day survival compared with prednisolone (75% vs. 56.6%; HR 0.528; *p* = 0.044) and markedly reduced infection‐related deaths (3.6 vs. 19.3%) [185]. A 2025 meta‐analysis confirmed improved short‐term survival with FMT, although the benefit attenuated by 6–12 months, and nearly all trials to date were conducted in India with naso‐duodenal delivery [186]. Phase 1/2 trials of lyophilized encapsulated FMT (for example, PRIM‐DJ2727; NCT05006430) and of FMT versus corticosteroids in steroid‐ineligible patients (NCT05285592) are underway in Western populations [181]. By contrast, broad‐spectrum gut decontamination has not improved survival: the Phase 3 AntibioCor trial (*n* = 292) found no survival benefit of adding amoxicillin–clavulanate to prednisolone and the nonabsorbable antibiotic rifaximin reduced infections and acute‐on‐chronic liver failure but not mortality in the RIFA‐AH pilot study [187, 188]. Probiotics (for example, Lactobacillus rhamnosus GG; NCT05178069) remain in early testing [189, 190].

*Anti‐Inflammatory and Antioxidant Agents*. IL‐1 blockade illustrates both the potential and the limitations of pathway‐specific immunomodulation: anakinra plus pentoxifylline and zinc showed no benefit in a randomized trial and a subsequent trial of anakinra plus zinc versus prednisone (NCT04072822) was stopped early after an interim analysis suggested inferior 90‐day survival with the anakinra regimen (70 vs. 90%) [113, 191]. The IL‐1β monoclonal antibody canakinumab was safe and showed a trend toward histological improvement, but did not improve clinical outcomes [192]. Among antioxidants, NAC combined with prednisolone improved 1‐month (8 vs. 24%) but not 6‐month survival in a landmark trial (NCT00863785), and a Phase 3 confirmatory trial is ongoing (NCT03069300) [193]. Metadoxine improved 3‐ and 6‐month survival in a single‐center open‐label study (NCT02161653) but requires multicenter validation [194].

*Bile Acid Signaling and FXR*. FXR agonism is mechanistically attractive given alcohol's disruption of the intestinal bile acid–FXR–FGF15/19 axis, but the first‐in‐class agent obeticholic acid was terminated early (NCT02039219) over safety concerns [195]. INT‐787, a next‐generation FXR agonist with an improved pharmacological profile, is now being evaluated in the Phase 2a FRESH trial (NCT05639543) [181].

Other exploratory therapies in early clinical testing include plasma exchange (NCT06355726), the TLR4 inhibitor TAK‐242 (NCT04620148), bovine colostrum and the colostrum‐derived preparation IMM‐124E (NCT02473341; NCT01968382), repurposed digoxin (NCT05014087), hyaluronic acid 35 (NCT05018481), omega‐5 fatty acids (NCT03723586), extended low‐dose prednisolone (NCT06155760), and intensive nutritional interventions (NCT06131177; target *n* = 2000) [181].

*Treating the Underlying AUD and the Role of Transplantation*. Treatment of the underlying AUD remains the intervention with the greatest long‐term impact. Baclofen and acamprosate are safe in advanced liver disease, yet AUD pharmacotherapy is prescribed to fewer than 2% of patients with ALD; integrating addiction care into hepatology practice is a stated priority of current guidelines [1, 13]. For steroid nonresponders, early liver transplantation yields excellent survival in carefully selected patients with prospective data confirming durable outcomes under stringent selection and follow‐up [196, 197].

#### 5.7.2 Lessons from Failed and Negative Clinical Trials

The failures of the past two decades are as instructive as the successes. Seven lessons emerge.

First, broad nonselective immunosuppression is hazardous in AH. The anti‐TNF agents infliximab and etanercept both increased infectious complications and mortality when combined with corticosteroids, a lesson reinforced by the excess of serious infections with prednisolone itself in STOPAH (13 vs. 7%) [198, 199]. Future therapies should modulate specific inflammatory pathways while preserving host defense, an inherent advantage of epithelial‐restricted agents such as IL‐22.

Second, the therapeutic window matters. Once severe AH progresses to multiorgan failure, liver‐directed therapies may provide limited benefit; regenerative agents (F‐652, G‐CSF) have enrolled predominantly patients with MELD ≤28, and larsucosterol's subgroup signals were strongest in less severely ill cohorts. Earlier disease stages—moderate AH and early cirrhosis—deserve dedicated trials rather than extrapolation from end‐stage populations [172].

Third, clinical heterogeneity can obscure treatment effects in unstratified trials. G‐CSF succeeded in Indian trials but failed in a German multicenter study; AHFIRM was negative globally but positive in the US subgroup. Differences in baseline severity, acute‐on‐chronic liver failure prevalence, infection burden, nutrition, and background care can obscure true drug effects. Biomarker‐guided enrichment (for example, MELD strata, Lille dynamics, or molecular signatures) and harmonized standards of care are prerequisites for future success [179, 184].

Fourth, endpoint selection remains unresolved. Prednisolone and NAC each improved early (28‐day or 1‐month) survival without durable benefit at 90 days or 6 months, whereas mortality endpoints may be underpowered in moderate disease [193]. Validated surrogate endpoints—histology, dynamic biomarkers—and patient‐reported outcomes deserve regulatory alignment, as piloted by the histology‐based canakinumab trial [192].

Fifth, failure to treat the underlying AUD limits durable benefit. In the STOPAH follow‐up, only a minority of survivors engaged in alcohol rehabilitation, and relapse drove rehospitalization; no pivotal AH trial before AlcHepNet integrated AUD treatment. Composite liver–abstinence endpoints, as adopted by AlcHepNet, should become standard [181].

Sixth, single‐pathway monotherapy is unlikely to suffice in a disease of convergent mechanisms. Pentoxifylline, selonsertib (NCT02854631), emricasan (NCT01912404), obeticholic acid (NCT02039219), and anakinra‐based regimens each targeted a single node and failed [113]. Rational combinations (corticosteroid plus NAC; TAK‐242 plus G‐CSF) and pleiotropic agents (larsucosterol) warrant further evaluation [191, 200].

Seventh, microbiome interventions require ecological precision rather than decontamination. Broad antibiotics provided no benefit in AntibioCor, whereas ecosystem‐restoring FMT improved survival; donor selection, delivery route, and durability of engraftment are important determinants of efficacy and safety [185, 187].

#### 5.7.3 Future Directions

Looking forward, we identify five priorities. First, biomarker‐guided, disease‐stage‐specific trial design should replace undifferentiated recruitment. Second, AUD pharmacotherapy and behavioral care should be routinely integrated into AH trials and clinical practice, as pioneered by the AlcHepNet platform. Third, rational combination regimens informed by mechanism—pairing anti‐inflammatory, regenerative, and gut‐directed agents—should be prioritized. Fourth, preclinical targets highlighted in this review, including ferroptosis and cuproptosis inhibition, noncoding RNA‐based therapeutics, and mitochondrial protectants, should be advanced into first‐in‐human studies. Fifth, global trial networks with harmonized standards of care and endpoints are needed to overcome the geographic heterogeneity that has confounded past studies. The ongoing Phase 3 larsucosterol trial, the enrolling AlcHepNet platform, and the broader early‐phase pipeline should clarify which strategies can provide durable clinical benefit.

## 6 Conclusion

ALD is an increasing global health burden for which pharmacological options remain limited. This review integrates epidemiology, pathogenesis, emerging therapeutic targets, and clinical translation, with particular emphasis on reciprocal interactions between gut dysbiosis and alcohol metabolism, oxidative stress, immune activation, and regulated cell death. By considering bacterial, fungal, and viral components together, it places microbiome changes within the broader network of host injury pathways rather than treating them as isolated observations.

Mechanistically, ALD arises from interactions among disordered ethanol metabolism, oxidative stress, innate immune activation, regulated cell death, and gut–liver axis dysfunction. Bacterial, fungal, and viral dysbiosis can intensify each of these processes. In turn, inflammation and hepatocyte death further impair intestinal barrier integrity and microbial homeostasis, establishing a self‐reinforcing cycle that promotes disease progression.

Clinical management remains primarily supportive and includes abstinence, nutritional therapy, corticosteroids in selected patients, infection control, and early liver transplantation for eligible patients with severe AH. Mechanism‐based therapies remain an unmet need. Strategies targeting the microbiota, inflammasome–pyroptosis pathways, bile acid signaling, and epithelial repair have shown activity in preclinical models or early clinical studies, but they require validation in larger trials with biomarker‐guided patient selection.

Several priorities warrant further investigation. Longitudinal human studies are needed to track changes in the microbiome, metabolome, and host immune response across the disease spectrum and to distinguish causal drivers from secondary consequences. Integrating metagenomics, metabolomics, proteomics, and single‐cell transcriptomics with machine learning may identify predictive biomarkers and molecular subtypes that support individualized treatment. In addition, clinical translation of FMT, phage therapy, and postbiotics requires standardized protocols for donor screening, engraftment monitoring, and safety assessment, particularly in patients with advanced liver disease and a high risk of infection. Multidisciplinary studies with harmonized endpoints and biomarker‐enriched trial designs will be essential for advancing precision treatment of ALD.

In conclusion, ALD is a preventable but increasingly prevalent disease driven by interconnected mechanisms centered on the gut–liver axis. Defining the bidirectional relationships between dysbiosis, oxidative stress, inflammation, and cell death may reveal stage‐specific therapeutic targets. Future research should prioritize longitudinal human studies, improved experimental models, mechanism‐enriched clinical trials, and integrated care that combines alcohol abstinence, nutritional support, and targeted therapy. These approaches may shift ALD management from late‐stage rescue toward earlier, mechanism‐based prevention and treatment.

## Author Contributions

Shuyan Wang and Yirui Hu drafted and revised the manuscript. Huikuan Chu supervised the study concept, revised the manuscript critically, and approved the final version. All authors contributed to the intellectual content of the review.

## Funding

This study was funded by the National Key R&D Program of China (No. 2022YFA1305600 to H.C.) and the National Natural Science Foundation of China (Nos. 82470584 and 82000561 to H.C.).

## Conflicts of Interest

The authors declare no conflicts of interest.

## Ethics Statement

The authors have nothing to report.

## Data Availability

Data sharing is not applicable to this review because no new datasets were generated or analyzed.
